# Investigator- and Site-Level Outcomes of Participation in an ED-Based Clinical Trial

**DOI:** 10.1001/jamanetworkopen.2025.55847

**Published:** 2026-02-09

**Authors:** Joseph E. Carpenter, Kathryn F. Hawk, Andrew Herring, Ethan Cowan, Ryan McCormack, Patricia H. Owens, Shara H. Martel, E. Jennifer Edelman, Kristen Huntley, Jeanmarie Perrone, Gail D’Onofrio

**Affiliations:** 1 Department of Emergency Medicine, Emory University School of Medicine, Atlanta, Georgia; 2 Department of Emergency Medicine, Yale School of Medicine, New Haven, Connecticut; 3 Department of Emergency Medicine, Highland Hospital, Oakland, California; 4 Department of Emergency Medicine, Rutgers New Jersey Medical School, Newark; 5 Department of Emergency Medicine, NYU Langone Medical Center, New York, New York; 6 Department of Internal Medicine, Yale School of Medicine, New Haven, Connecticut; 7 National Institute on Drug Abuse, Rockville, Maryland; 8 Department of Emergency Medicine, Perelman School of Medicine at the University of Pennsylvania, Philadelphia

## Abstract

This survey study examines outcomes related to training, clinical practice, professional advancement, and institutional change among sites participating in a clinical trial evaluating emergency department (ED) buprenorphine.

## Introduction

Randomized clinical trials (RCTs) typically report patient-oriented outcomes. However, site engagement in RCTs requires substantial investment in personnel, training, and institutional resources, which may have lasting effects on personnel and institutional culture.^1^ Additionally, engagement in research has been associated with improved health care performance at the institutional level, including in prior studies at substance use treatment centers within the National Institute on Drug Abuse (NIDA) Clinical Trials Network (CTN).^2^ Nonetheless, little has been reported about downstream effects of site participation for emergency department (ED)-based studies, including investigator professional advancements and institution-wide improvements in clinical practice. We sought to measure individual- and site-level benefits associated with participation in the Emergency Department-Initiated Buprenorphine and Validation Network Trial (ED-INNOVATION).^3^

## Methods

This is a cross-sectional survey of site principal investigators (SPIs) who participated in ED-INNOVATION, a NIDA CTN-funded multicenter ED-based effectiveness and implementation study from March 2020 to October 2024, which (1) provided training and technical support to 33 geographically diverse academic EDs across the US to support ED-initiated buprenorphine treatment, and (2) conducted a 2000-patient RCT comparing the effectiveness of sublingual vs extended-release buprenorphine in engaging patients with opioid use disorder (OUD) into treatment at 7 days.^3^ Investigators employed implementation facilitation strategies^4^ to enhance the adoption of ED-initiated buprenorphine at study sites, which had to meet prespecified criteria prior to beginning RCT recruitment.^3^

Survey questions covered domains including training and adoption of buprenorphine in EDs, community engagement, institutional practice changes, professional advancement, and additional research opportunities (eAppendix in Supplement 1). Surveys were distributed via email in November 2024 and completed online in Qualtrics. Responses were both quantitative and free text. Quantitative results are presented descriptively. Free text was reviewed for common themes and illustrative quotes were selected. Results are reported according to the American Association for Public Opinion Research (AAPOR) reporting guideline for survey studies. This project was approved by the WCG institutional review board including a waiver of consent documentation.

## Results

Thirty-one SPIs completed the survey (response rate, 100%), representing 33 EDs across 23 states. Sites trained 3811 clinicians to provide buprenorphine (median [IQR], 110 [71-140]) and supported 373 clinical and research staff members (median [IQR], 11 [5-18]) across a range of disciplines (Figure). Implementation facilitation processes included engagement with community partners to inform the ED buprenorphine program implementation, developing site-specific practices and referral processes to ensure ongoing OUD treatment after discharge. Sites engaged a median of 6 community partners, most commonly opioid treatment programs, individual stakeholders, and outpatient clinics (Figure).

**Figure.**
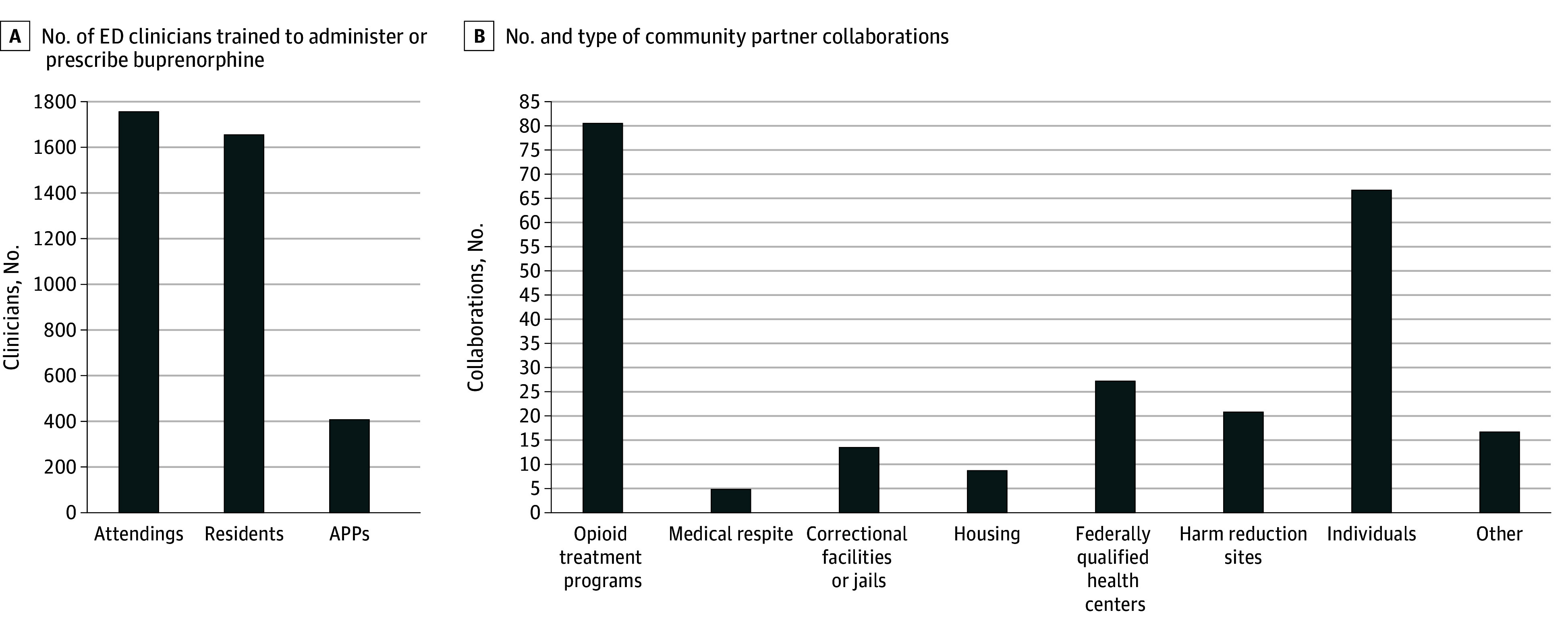
Clinical Practice and Community Collaborations Beyond the Clinical Trial APP indicates advanced practice provider; ED, emergency department.

SPIs reported positive effects across multiple institutional and individual domains (Table). Seven sites (21%) reported that the study helped deploy ED-based substance use navigators and 4 (12%) added extended-release buprenorphine to the hospital formulary. Two SPIs (6%) had expanded their efforts to additional EDs in the region, 1 (3%) with state funding. ED-INNOVATION provided training and/or support for 80 early-stage investigators (ESIs); 2 (3%) have received NIH career development (K) awards; 3 (4%) have received R01s; and 1 (1%) submitted a K23. Fifty-five investigators (69%) achieved board certification in addiction medicine; an additional 13 (16%) are board-eligible.

**Table.** Overall Themes

Theme	Quote
Institutional change and improved clinical practice	“Our ED faculty converted from 90% opposed to buprenorphine initiation to 90% in favor of treatment initiation for patients with OUD. In addition, we were able to train 80% of attendings and 90% of APPs to obtain their X-waivers.”
	“This study completely transformed the culture of our ED. We went from an ED that essentially never treated opioid withdrawal…to one in which nearly everyone was X-waivered and now to a place where we have ED physicians doing outpatient addiction medicine, a methadone dosing program in the ED, and a culture that OUD is a disease to be treated as any other.”
Research capacity	“Our hospital seems to have caught the research bug and will be looking into other clinical trials we can get involved in. I have seen our RAs [progress] their careers into more advanced involvement in research roles in private and academic settings.”
Professional advancement	“[Our team was] involved with 3 other CTN Trials as site PIs or MCs because of the support and experience from CTN-0099. In addition, we were a part of a PCORI clinical trial that is ongoing. The experience and support from CTN-0099 has helped to inform other research questions and improved collaborations with the addiction medicine community.”
	“[Investigator] received extensive training as site PI in how to conduct an RCT. He gained critical experience in how to manage a team of research assistants and gained skills in program implementation and optimization from experienced investigators. He also used this opportunity to develop meaningful relationships throughout the university and medical center, which provided a foundation for his career moving forward.”
	“Participating in NIDA CTN-0099 as a site PI proved to be invaluable in my own career development, as I learned a great deal about managing a large multi-site trial, including optimal design considerations, participant recruitment and retention strategies, and responding to unanticipated trial obstacles.”
Downstream effects	“I now direct a system-wide initiative to provide ED-initiated buprenorphine and harm reduction services (take-home naloxone, fentanyl test strips) to the 11 hospitals”.
	“ED-initiated buprenorphine is being sustained through a state-funded grant called MOUD Access, and we are continuing to expand the number of EDs involved (currently at 10). We are also trying to support lower volume hospitals with substance use navigators via telemedicine to assist with ED initiated buprenorphine.”

Abbreviations: APP, advanced practice provider; ED, emergency department; MC, medical clinician; NIDA, National Institute on Drug Abuse; OUD, opioid use disorder; PI, principal investigator; RCT, randomized clinical trial.

## Discussion

Improved outcomes across the domains of training, clinical practice, professional advancement, community engagement, and institutional change were associated with site engagement in the NIH funded ED-INNOVATION Effectiveness-Implementation trial. Enhancing clinical practice and professional development on this scale would typically require large investment by health care and/or government organization. The association reported here provides additional evidence of the considerable return on investment for NIH-funded research, calculated to be $2.56 for every $1 spent as of March 2025.^5^

Emergency medicine (EM) ranks last among clinical specialties in the percentage of full-time faculty members with NIH funding as PI.^6^ Our findings suggest that engaging ESIs in large clinical trials like ED-INNOVATION may expand the EM research pipeline.

Limitations include potential for recall and social desirability bias. The short timeframe of data collection limited measurement of long-term outcomes. We did not evaluate secular trends in ED buprenorphine training and practice improvements. Some outcomes may have occurred directly or secondarily to protocol compliance.

In this survey study of sites engaged in a large multicenter clinical trial implementing and evaluating ED buprenorphine, we observed benefits beyond the clinical trial representing a broad range of individual and institutional advancements associated with site engagement. This included increased training, improved clinical practice, community engagement, and career development opportunities for ESIs.

## Notes

Supplement 1. eAppendix. Yale Qualtrics survey tool

Supplement 2. Nonauthor Collaborators

Supplement 3. Data Sharing Statement
