# Supplementary material for: Investigator- and Site-Level Outcomes of Participation in an ED-Based Clinical Trial
Source: JAMA Netw Open. 2026 Feb 9;9(2):e2555847. doi: 10.1001/jamanetworkopen.2025.55847 (PMC12887738; doi:10.1001/jamanetworkopen.2025.55847)
Supplement: Supplement 1. — eAppendix. Yale Qualtrics survey tool [file jamanetwopen-e2555847-s001.pdf]

Supplemental Online Content

Carpenter JE, Hawk KF, Herring A, et al; on behalf of the ED-INNOVATION Investigators. Investigator- and site-level outcomes of participation in an ED-based clinical trial. *JAMA Netw Open.* 2026;9(2):e2555847. doi:10.1001/jamanetworkopen.2025.55847

eAppendix. Yale Qualtrics survey tool

This supplemental material has been provided by the authors to give readers additional information about their work.

© 2026 Carpenter JE et al. *JAMA Network Open*.

eAppendix. Yale Qualtrics survey tool

Yale Qualtrics Survey Tool

Intro. Please enter your name and site.

[ ]

Q1. Number clinical/research sites involved in the research (If more than your clinical site please note)

[0]

Clinical/Research Sites: (Specify)

[ ]

Q2. Number of clinicians trained to initiate, administer, or prescribe buprenorphine.

Attending	0
Residents	0

© 2026 Carpenter JE et al. *JAMA Network Open*.

APPS	0
Other: (Specify)	0
Total	0

Q3. Number of community partners engaged to provide input to inform research effort (Organizations and or Individuals)

Correctional Facilities/Jails:	0
Medical Respite:	0
Community Opioid Treatment Program (OTP)	0
Housing:	0
FQHC or other clinics: (Specify)	0
Harm Reduction Sites: (Specify)	0

© 2026 Carpenter JE et al. *JAMA Network Open*.

Other: (Specify)

	0

Individuals: (Specify profession)

	0

Total	0

Q4. Number of community partners research evidence was disseminated or communicated to. (Organizations and or Individuals)

Correctional Facilities/Jails	0
Medical Respite	0
Community Opioid Treatment Program (OTP)	0
Housing	0

FQHC or other clinics: (Specify)

	0

© 2026 Carpenter JE et al. *JAMA Network Open*.

Harm Reduction Sites: (Specify)

[ ]	0

Other: (Specify)

[ ]	0

Individuals: (Specify profession)

[ ]	0

Total	0

Q5. Number of study publications to date. (Only applicable to Yale Site)

Publications	0
Total	0

© 2026 Carpenter JE et al. *JAMA Network Open*.

Q6. Number of presentations or posters at professional meetings.

0 Posters/Presentations: (Specify)

Q7. Number of early stage investigators trained or supported. (Include a few sentences on they are and how they were trained)

Early Stage Investigators

0

Total 0

© 2026 Carpenter JE et al. *JAMA Network Open*.

Q8. Number of research and clinical staff trained/employed/supported by study.

Research Assistants	0
Other Co-Investigators	0
Medical Clinicians	0
Peers	0
Substance Use Navigators	0
Other: (Specify)	0
Total	0

Q9. Number of dissemination resources developed (intervention manuals, infographics, podcasts, webinars, etc.) Please note the title and approximate date.

0 Disseminations Resources:

© 2026 Carpenter JE et al. *JAMA Network Open*.

Q10. Any EHR modifications that you have spearheaded, pathways etc

Q11. Number of physicians that have been boarded in Addiction Medicine and number approved to site on the boards.

0	Boarded physicians
0	Approved to sit for the boards

© 2026 Carpenter JE et al. *JAMA Network Open*.

Q12. Quotes or stories from the study that could highlight the importance or impact of the research. This could include quotes from patients, peer coaches, clinicians, researchers, community partners, etc.

Q13. How has this CTN research impacted care of patients in your community? (Information not already included above.)

© 2026 Carpenter JE et al. *JAMA Network Open*.

Q14. How are research interventions being sustained and/or scaled up at a local, regional, or national level?

Powered by Qualtrics

© 2026 Carpenter JE et al. *JAMA Network Open*.
