# Supplementary material for: Investigator- and Site-Level Outcomes of Participation in an ED-Based Clinical Trial
Source: JAMA Netw Open. 2026 Feb 9;9(2):e2555847. doi: 10.1001/jamanetworkopen.2025.55847 (PMC12887738; doi:10.1001/jamanetworkopen.2025.55847)
Supplement: Supplement 2. — Nonauthor Collaborators [file jamanetwopen-e2555847-s002.pdf]

Supplemental Online Content: Nonauthor Collaborators
*First name, last name, and suffix (if applicable) are required and will appear in PubMed.

***Group Name(s): The ED-INNOVATION investigators**

***First Name and Middle Initial(s)**	***Last Name**	***Suffix (eg, Jr, III)**	Academic Degrees	Institution	Location (city, state/province, country)	Role or Contribution, eg, chair, principal investigator	Group (if more than 1 Group listed in the byline) and/or Subgroup (eg, Steering Committee)
Evan	Schwarz		MD	Barnes Jewish Hospital	St. Louis, MO	Principal Investigator	
Brandt	Williamson		MD	Berkeley Medical Center	Martinsburg, WV	Principal Investigator	
Jon	Lee		MD	Berkeley Medical Center	Martinsburg, WV	Co-Investigator	
Christopher	Jones		MD	Cooper Health	Camden, NJ	Principal Investigator	
Patricia	Lanter		MD, MS	Dartmouth Hitchcock Medical Center	Lebanon, NH	Principal Investigator	
Jennifer	Pope		MD	Dartmouth Hitchcock Medical Center	Lebanon, NH	Co- Investigator	
Andrew	King		MD	Detroit Receiving Hospital	Detroit, MI	Principal Investigator	
Joseph	Carpenter		MD	Grady Memorial Hospital	Atlanta, GA	Principal Investigator	
Lauren	Whiteside		MD, MS	Harborview Medical Center	Seattle, WA	Principal Investigator	
Herbert	Duber		MD, MPH	Harborview Medical Center	Seattle, WA	Co-Investigator	
James	Miner		MD	Hennepin County Medical Center	Minneapolis, MN	Principal Investigator	
Jon	Cole		MD	Hennepin County Medical Center	Minneapolis, MN	Co-Investigator	
Jacob	Manteuffel		MD	Henry Ford Hospital	Detroit, MI	Principal Investigator	
Joseph	Miller		MD	Henry Ford Hospital	Detroit, MI	Co-Investigator	
Andrew	Herring		MD	Highland Hospital	Oakland, CA	Principal Investigator	
Andrew	Stolbach		MD, MPH	Johns Hopkins Hospital	Baltimore, MD	Principal Investigator	
Michael	Baumann		MD	Maine Medical Center	Portland, ME	Principal Investigator	
Tania	Strout		PhD, RN, MS	Maine Medical Center	Portland, ME	Co-Investigator	
Lindsey	Jennings		MD, MPH	Medical University of South Carolina	Charleston, SC	Principal Investigator	
Bernard	Chang		MD, PhD	New York Presbyterian Hospital	New York, NY	Principal Investigator	
Howard	Kim		MD	Northwestern Memorial Hospital	Chicago, IL	Principal Investigator	
Jeanmarie	Perrone		MD	Penn Presbyterian Medical Center	Philadelphia, PA	Principal Investigator	
David	Jang		MD, MSc	Penn Presbyterian Medical Center	Philadelphia, PA	Co-Investigator	
Eric	Ketcham		MD, MBA	Presbyterian Hospital	Albuquerque, NM	Principal Investigator	
Elizabeth	Samuels		MD, MPH, MHS	Rhode Island Hospital and The Miriam Hospital	Providence, RI	Principal Investigator	
Rachel	Wightman		MD	Rhode Island Hospital and The Miriam Hospital	Providence, RI	Principal Investigator	
Erik	Anderson		MD	San Leandro Hospital	San Leandro, CA	Principal Investigator	
Jason	Wilson		MD, PhD	Tampa General Hospital	Tampa, FL	Principal Investigator	
Joseph	D’Orazio		MD	Temple Health	Philadelphia, PA	Principal Investigator	
Paul Quincy	Moore		MD	University of Chicago Medicine	Chicago, IL	Principal Investigator	
Cameron	Crandall		MD	University of New Mexico Hospital	Albuquerque, NM	Principal Investigator	
James	Cotton		MD	University of New Mexico Hospital	Albuquerque, NM	Co-Investigator	
Michael	Lynch		MD	University of Pittsburgh Medical Center	Pittsburg, PA	Principal Investigator	
Gerald	Cochran		MSW, PhD	University of Utah Hospital	Salt Lake City, UT	Co-Investigator	

Supplemental Online Content: Nonauthor Collaborators
*First name, last name, and suffix (if applicable) are required and will appear in PubMed.

***First Name and Middle Initial(s)**	***Last Name**	***Suffix (eg, Jr, III)**	Academic Degrees	Institution	Location (city, state/province, country)	Role or Contribution, eg, chair, principal investigator	Group (if more than 1 Group listed in the byline) and/or Subgroup (eg, Steering Committee)
Troy	Madsen		MD	University of Utah Hospital	Salt Lake City, UT	Principal Investigator	
Peter	Taillac		MD	University of Utah Hospital	Salt Lake City, UT	Principal Investigator	
Alyrene	Dorey		MD	University of Utah Hospital	Salt Lake City, UT	Principal Investigator	
Ross	Sullivan		MD	Upstate University Hospital	Syracuse, NY	Principal Investigator	
Tyler	Barrett		MD	Vanderbilt University Medical Center	Nashville, TN	Principal Investigator	
Amanda	Wilson		MD	Vanderbilt University Medical Center	Nashville, TN	Principal Investigator	
Kathryn	Hawk		MD, MHS	Yale New Haven Hospital	New Haven, CT	Principal Investigator	
