# Supplementary material for: Investigator- and Site-Level Outcomes of Participation in an ED-Based Clinical Trial
Source: JAMA Netw Open. 2026 Feb 9;9(2):e2555847. doi: 10.1001/jamanetworkopen.2025.55847 (PMC12887738; doi:10.1001/jamanetworkopen.2025.55847)
Supplement: Supplement 3. — Data Sharing Statement [file jamanetwopen-e2555847-s003.pdf]

Data Sharing Statement

Carpenter. Individual- and Site-Level Outcomes of Participation in an ED-Based Clinical Trial. *JAMA Netw Open*. Published February 09, 2026. doi:10.1001/jamanetworkopen.2025.55847

Data
Data available: No

Additional Information
Explanation for why data not available: Data will be provided to qualified investigators upon request.
